# Filtering and storage working memory networks in younger and older age

**DOI:** 10.1002/brb3.544

**Published:** 2016-09-14

**Authors:** Anne‐Katrin Vellage, Andreas Becke, Hendrik Strumpf, Bernhard Baier, Mircea Ariel Schönfeld, Jens‐Max Hopf, Notger G. Müller

**Affiliations:** ^1^ Neuroprotection GroupGerman Centre of Neurodegenerative DiseasesMagdeburgGermany; ^2^ Institute of Cognitive Neurology and Dementia ResearchMagdeburgGermany; ^3^ Leibniz Institute for NeurobiologyMagdeburgGermany; ^4^ Department of NeurologyEdith‐Stein ClinicBad BergzabernGermany; ^5^ Department of NeurologyOtto von Guericke UniversityMagdeburgGermany; ^6^ Center of Behavioral Brain Sciences (CBBS)MagdeburgGermany

**Keywords:** fMRI, healthy aging, prefrontal cortex, selective attention, working memory

## Abstract

**Introduction:**

Working memory (WM) is a multi‐component model that among others involves the two processes of filtering and storage. The first reflects the necessity to inhibit irrelevant information from entering memory, whereas the latter refers to the active maintenance of object representations in memory. In this study, we aimed at a) redefining the neuronal networks sustaining filtering and storage within visual working memory by avoiding shortcomings of prior studies, and b) assessing age‐related changes in these networks.

**Methods:**

We designed a new paradigm that strictly controlled for perceptual load by presenting the same number of stimuli in each of three conditions. We calculated fMRI contrasts between a baseline condition (low filter and low storage load) and conditions that posed high demands on filtering and storage, respectively, in large samples of younger (*n *= 40) and elder (*n *= 38) participants.

**Results:**

Our approach of comparing contrasts between groups revealed more extensive filter and storage WM networks than previous studies. In the younger group, filtering involved the bilateral insulae, the right occipital cortex, the right brainstem, and the right cerebellum. In the elder group, filtering was associated with the bilateral insulae, right precuneus, and bilateral ventromedial prefrontal cortex. An extensive neuronal network was also found during storage of information in the bilateral posterior parietal cortex, the left ventromedial prefrontal cortex, and the right precuneus in the younger participants. In addition to these brain regions, elder participants recruited the bilateral ventral prefrontal cortex, the superior, middle and inferior and temporal cortex, the left cingulum and the bilateral parahippocampal cortex.

**Conclusions:**

In general, elder participants recruited more brain regions in comparison to younger participants to reach similar accuracy levels. Furthermore, in elder participants one brain region emerged in both contrasts, namely the left ventromedial prefrontal cortex. Hence, elder participants seem to routinely recruit this brain region in demanding tasks, irrespective of whether filtering or storing is challenged.

## 1 Introduction

Visual working memory (VWM) provides an online workspace where information about complex visual scenes can be efficiently accessed and updated (Baddeley, 1986; Smith & Jonides, 1998). As VWM capacity is limited to a few (2–4) objects (Cowan, 2001; Duncan et al., 1999; Pashler, 1988; Vogel, Woodman, & Luck, 2001), attentional control mechanisms are necessary that prioritize the processing of relevant over irrelevant information (Kane, Bleckley, Conway, & Engle, 2001; Vogel, McCollough, & Machizawa, 2005). Indeed, it has been shown that the ability to filter out irrelevant information determines the individual VWM capacity (Vogel & Machizawa, 2004).

Regarding the neural substrates of the two processes filtering and storage, the former has been attributed to different brain areas and neural networks: McNab and Klingberg (2007) observed a frontostriatal network which sustains filtering of information; others attributed this function mainly to the thalamus (Baier, Kleinschmidt, & Müller, 2006; Bočková et al., 2011). As a storage node, the posterior parietal cortex (PPC) came to the fore because on the one hand VWM storage capacity is reflected in parietal activity (McNab & Klingberg, 2007; Todd & Marois, 2004, 2005; Vogel & Machizawa, 2004; Vogel et al., 2005; Xu & Chun, 2005); on the other hand, the PPC plays a major role in the dorsal attention control system (Corbetta & Shulman, 2002) rendering it also a potential candidate for information filtering. One potential source of this discrepancy is that many prior studies confounded perceptual and memory loads so that in case more items had to be stored and also if more items had to be perceptually processed. Therefore, one aim of this study was to reevaluate brain regions involved in filter and storage processes in an fMRI experiment using a delayed matching‐to‐sample paradigm in which the visual input was kept constant to unconfound memory from perceptual and attentional load effects.

A further aim of this study was to assess aging effects on filter and storage processes. During healthy aging impairments in WM and attention processes emerge which are likely caused by a decline of neurotransmitter function (Li & Rieckmann, 2014), loss of cortical thickening and metabolic activity. By conducting a meta‐analysis over 30 research reports that tested young and elder participants in a WM or inhibition task, Turner and Spreng (2012) created brain maps showing activity patterns that differed between age groups during the mentioned tasks. In WM tasks, elder group as compared to younger group showed decreased activation in inferior parietal sulcus, insula and frontal eye fields and increased activation in frontal brain areas, including the supplementary motor area and the inferior frontal gyrus. In inhibition tasks, elderly participants showed a decrease in activation in occipital and an increase in frontal brain areas only. The stronger frontal brain activity in both tasks in elderly was interpreted as a compensation mechanism (Reuter‐Lorenz & Cappell, 2008) reflecting a need for increased cognitive control.

Cognitive deficits seen in elder participants are often compared with deficits in children (Hasher & Zacks, 1988; Sander, Werkle‐Bergner, & Lindenberger, 2011). However, current findings reveal slight differences between the filter deficits observed in children and those observed in the elder. In an EEG study, it was shown that inhibition of irrelevant information is not abolished during aging but seems to become delayed resulting in longer response times (Gazzaley et al., 2008). These results were reaffirmed by a study of Jost, Bryck, Vogel, and Mayr (2011), who used a delayed matching‐to‐sample task in which relevant and irrelevant stimuli were presented in a single array instead of multiple ones as in the Gazzaley et al. (2008) study. Given that cognitive deficits in elderly resemble cognitive inabilities in children only on the surface, we decided to reevaluate the processes underlying filtering and storage in VWM in healthy young and elder participants. To that end, we developed a new paradigm in which filter and storage demands were modulated separately, while the visual input was kept constant, allowing an unbiased comparison of both processes.

## 2 Material and Methods

The data presented here are part of a larger project in which drug effects on attention and memory processes are assessed too. Here, only data from the placebo measurements are presented. As one portion of participants was randomized to receive the placebo first, whereas the others received the placebo in the second session; sequence (first or second) was included in the ANOVA as a between factor to control for training effects. The results were Greenhouse–Geisser corrected for nonsphericity, if necessary.

*Subjects:* Four participants (1 young, 3 elder) had to be excluded from the analysis as their hit rates in the fMRI task were below 0.6. A total of 40 younger (14 female, 26 male, mean age 25.7 years, range 21–32 years) and 38 elder (24 female, 14 male, mean age 65.8 years, range 58–74 years) right‐handed healthy and neurologically normal participants with normal or corrected‐to‐normal vision were recruited from an academic environment or via advertisements in local newspapers and included in the analysis. Both age groups were comparable in terms of gender distribution (x² = 2.558, *df* = 1; *p *= .110) and gender did not influence the observed fMRI effects in either age group.

The participants were paid volunteers and gave written informed consent before participation. The study was approved by the local ethics committee.

**Figure 1:**
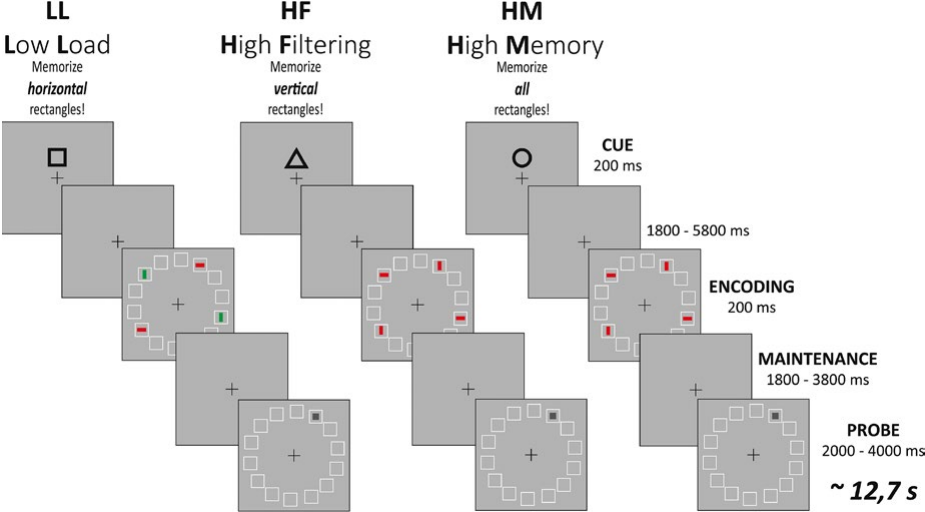
Schematic illustration of the experimental design. An instruction cue was followed by a sample display with 14 placeholder squares arranged in a circle. The squares were filled with either four red or two red and two green rectangles. After a second delay, a probe display was shown with a gray dot in one of the placeholder squares. Subjects had to decide by button press, whether the probe was shown in a position formerly occupied by a target or not

*VWM task:* The fMRI experiment included three conditions with the following demands: Baseline (low filtering and low storage, LL), high filtering (HF), and high memory storage (HM). In all three conditions, four colored rectangles were shown and only preceding cues indicated which task had to be performed (Fig. 1). Before the experiment started, participants were familiarized with the cues so that their meanings became clear.

This procedure aimed at leaving the perceptual input during memory encoding largely the same. The baseline condition, LL, started with the presentation of a square cue that indicated to the participants to memorize only the two horizontal rectangles in the target color (e.g., red). In addition, the memory array included two vertical rectangles in the other color (e.g., green). Hence, in the LL condition, participants could simply ignore objects based on their deviating color. A triangle cue marked the beginning of a HF trial, in which participants had to memorize the positions of two vertical rectangles while ignoring the two horizontal ones. In this condition, all rectangles were either in green or red color so that the selection had to be based on orientation. A circle cue marked the HM condition and indicated that the positions of all four upcoming rectangles had to be memorized. The memory array was followed by a delay and then by a probe stimulus (gray dot) to which participants had to decide by button press with the index or middle finger of their right hand, whether the probe location had been occupied by a target stimulus in the preceding memory array or not. When the probe stimulus was not in the position of a target, it was either on a position adjacent to a target or, in case distractors had been presented, with equal probability on a distractor position. The required responses (yes or no) were distributed evenly across all trials. Subjects completed six runs à 58 trials (348 trials in total) with one run lasting 9 min. At the beginning of each run, an instruction was shown that indicated which of the two rectangle colors was relevant for the upcoming run of trials. In half of the runs, participants had to attend the red rectangles, in the other half of the runs they had to attend the green rectangles. This was done to prevent a possible color bias. Before participating in the main experiment, all participants completed one short practice session (12 trials) outside the scanner.

Stimuli were presented against a gray background (luminance 41.2 cd m^−2^). Cue stimuli (0.6° × 0.6°) were presented 0.5° above a fixation cross that was placed in the center (16.4° from side, 18.8° from top) of the screen. Memory and probe stimuli appeared within fourteen task irrelevant placeholder squares (size 0.9° × 0.9°) arranged in a circle (diameter 7.3°, minimum difference squares 1.5° center to center). Each memory array contained two horizontal and two vertical rectangles (size 0.8° × 0.3°) which appeared in four of the placeholder squares. The memory stimuli consisted of two green and two red, four red or four green rectangles (luminance: red = 31 cd m^−2^; green = 34 cd m^−2^). The probe stimuli contained a gray square (size 0.3° × 0.3°) which appeared in one of the placeholder squares. The instruction cues were presented for 0.2 s and were followed by a delay of 1.8, 3.8 or 5.8 s. The memory array was presented for 0.2 s and was followed by a delay of 1.8 or 3.8 s. All trials ended with a probe stimulus that lasted for 1.4 s and was followed by a delay of 0.6 or 2.6 s.

### 2.1 Behavioral data analysis

We calculated storage and filter scores from the hit rates. Differences between performance in the LL and HM condition are referred to as storage score with large values indicating impairment with increasing memory load, that is, a storage deficit. The filter score was assessed by calculating the difference between the LL and the HF condition. In the case of distractors being unnecessarily stored (filtering deficit), performance in the HF condition should be low leading to a higher filter score. This subtracting procedure eliminates possible baseline differences in performance across groups (Baier et al., 2010). An ANOVA was carried out on filter and storage scores, including the between factor sequence to account for the order of measurement (first or second session).

#### 2.1.1 fMRI data acquisition

Because the MR scanner which had been used in the younger group (Siemens Trio) was no longer available when data acquisition started in the elder participants, they were investigated in a different machine (Siemens Verio) whereby scanning protocols were kept as comparable as possible. Furthermore, our approach of calculating contrasts within groups should minimize potential activation differences due to the usage of different machines. Nevertheless, with the fMRI data, we renounced calculating statistic comparisons between groups.

#### 2.1.2 Younger group

A 3T Siemens Magnetom Trio syngo MR A35 scanner (Erlangen, Germany) equipped with an eight‐channel head coil was used to measure blood oxygenation level‐dependent (BOLD) brain activity in the younger groups. Stimuli were back‐projected by a LCD projector on a screen positioned behind the coil. The screen was viewed by the participants via a mirror attached to the head coil. Functional images were acquired with a T2*‐weighted echo planar imaging (EPI) gradient echo sequence (FoV 224 × 224 mm, voxel size = 3.5 × 3.5 × 3.5 mm, TR = 2000 ms, TE = 29 ms, flip angle = 80° in an odd‐even interleaved sequence. Thirty‐four 3.5 mm thick axial slices (64 mm × 64 mm in plane, no gap) parallel to the AC‐PC line were acquired for 255 volumes in each run. Whole‐head T1‐weighted images were collected with an MP‐RAGE sequence (96 sagittal slices, thickness = 2 mm, FoV 256 × 256 mm, no gap, spatial resolution = 1 × 1 × 2 mm, TR = 1650 ms, TE = 5.01 ms, TI = 1100 ms).

#### 2.1.3 Elder group

A 3T Siemens Magnetom Verio syngo MR B19 scanner (Erlangen, Germany) equipped with a 32‐channel head coil was used in the elder group. The same projection system as in the younger group was used to present stimuli. Functional images were collected using 32 axial slices (64 mm × 64 mm in plane, no gap) covering the whole brain with a T2*‐weighted EPI gradient echo sequence in an odd‐even interleaved sequence (FoV 224 × 224 mm, voxel size = 3.5 × 3.5 × 3.5 mm, TR = 2000 ms, TE = 38 ms, flip angle = 80°). Axial slices were acquired parallel to the AC‐PC line for 255 volumes in each run. Wholehead T1‐weighted images were collected with an MP‐RAGE sequence (96 sagittal slices, thickness = 2 mm, FoV 256 × 256 mm, no gap, spatial resolution = 1 × 1 × 2 mm, TR = 1660 ms, TE = 5.05 ms, TI = 1100 ms).

#### 2.1.4 fMRI data analysis

All data were processed with the SPM8 software package (Welcome Department of Cognitive Neurology, University College London, UK; RRID: SCR_007037) and MATLAB R2009b (The Mathwork Inc.), which included slice time correction, realignment to the first volume, coregistration to the individual anatomical images, normalization to the Montreal Neurological Institute (MNI) template (Friston et al., 1995) and resampling into a voxel size of 3 × 3 × 3 mm³. Spatial normalized images were smoothed with an isotropic 6‐mm FWHM Gaussian kernel and high pass filtered (cut‐off 128 s). Global scaling was applied across an individual session to remove global signal drifts before GLM analysis. No participants had to be excluded due to excessive head motion (more than 5 mm).

Blood oxygenation level‐dependent responses were modeled by delta functions at the time of stimulus onsets. For each individual, the time courses of the hemodynamic BOLD responses in the different conditions (LL, HF, and HM) were analyzed at the voxel level using a linear regression model that yielded separate time courses (12 time points (seconds) in each time course) for the cue phase, encoding phase and response phase of each condition. The movement parameters derived from the realignment process were included as covariates into the model (Friston et al., 1998) as well as all trials in which the participants made a wrong response leading to 16 regressors in total for each run (LL, HF and HM × cue, × encoding, × response phase, errors, 6 × movement). To identify regions activated by attentional filtering and memory storage, respectively, we calculated different contrasts for each subject and each session individually for each condition in the encoding phase (LL, HF, and HM) in a first‐level analysis and used the contrast images of every subject in a second‐level analysis. First, an ANOVA including the within factor task (three levels: LL, HF, and HM) and the between factor group (two levels: first and second) was carried out and then the contrast HF > LL was calculated to identify filter‐related areas and the contrast HM > LL to reveal storage‐related brain regions. The minimal distance between cluster peaks was set at 18 mm. Second, we conducted a conjunction analysis across both contrasts (filter and storage contrast) for both age cohorts (younger and elder) separately. Including age as a regressor did not change results significantly. In all analyses, the cut‐off value was set to *p *< .001 uncorrected like in many prior studies (e.g., Groussard et al., 2010; Van de Sand, Sprenger, & Büchel, 2015).

## 3 Results

### 3.1 Behavior

**Figure 2:**
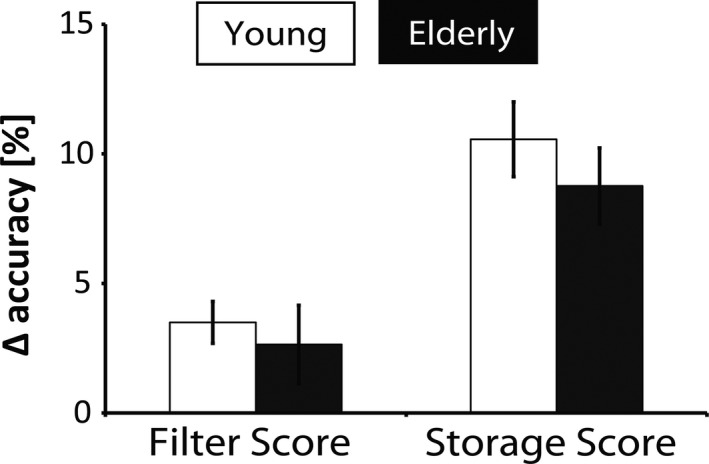
Filter scores (hit rates in the LL minus the HF condition) and storage scores (hit rates in the LL minus the HM condition). Error bars indicate the standard error of the mean. The scores did not differ significantly between young and old participants

Behavioral data are presented in Fig. 2. A multivariate ANOVA with the filter and storage accuracy scores revealed neither a significant difference between age groups in the filter (*F*_1,73_ = 0.252, *p *= .617) nor in the storage score (*F*_1,73_ = 0.867, *p* = .355). Similarly, no age effects were observed when instead of difference scores, hits and correct rejections were directly assessed. Response times were generally longer in the group of elder participants. However, this occurred independently from the specific task, and therefore, data are not presented here.

### 3.2 fMRI data

#### 3.2.1 Filtering

**Figure 3:**
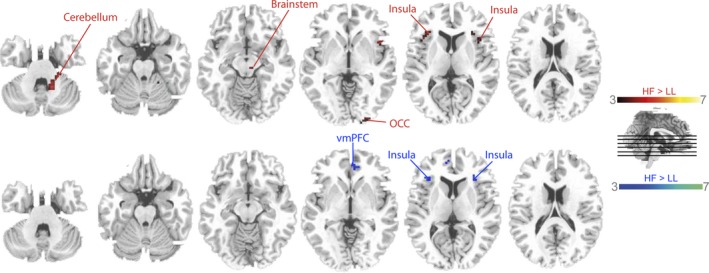
Filter contrast. Colored voxel mark a significant contrast HF > LL for young (red) and old participants (blue), *p *= .001 (uncorr.)

In order to address the attentional filtering network, we looked which areas were more active during the condition with high filtering demands (HF) than during the condition in which less filtering was required (LL). In the group of younger participants, this contrast revealed the bilateral insulae, the right occipital cortex (OCC), the right brainstem and the right cerebellum at a significance level of *p *< .001 (Fig. 3). Similar to younger participants, elder participants recruited the bilateral insulae as well (Fig. 3). In addition, the left ventromedial prefrontal cortex (vmPFC) and the right precuneus were found during filtering. Peaks of all clusters are reported in Table 1.

**Table 1:** Peak activations for the filter contrast (HF > LL)

Anatomical structure	Hemisphere	MNI coordinates (x, y, z)			Max. T‐value	Cluster size
Young						
Insula	R	48	14	−5	4.47	51
	R	33	23	4	3.51	
	L	−33	26	4	3.94	16
OCC	R	30	−100	−2	4.04	23
Brainstem	R	6	−25	−8	4.70	11
Cerebellum	R	15	−52	−35	4.45	38
	R	24	−34	−38	4.36	
Old						
Insula	R	36	26	7	4.97	22
	L	−30	20	10	4.26	37
dPFC	–	0	56	1	3.72	20
	L	−9	41	−5	4.02	23
Precuneus	R	12	−52	40	4.03	11

L, left; R, right; OCC, occipital cortex.

#### 3.2.2 Storage

**Figure 4:**
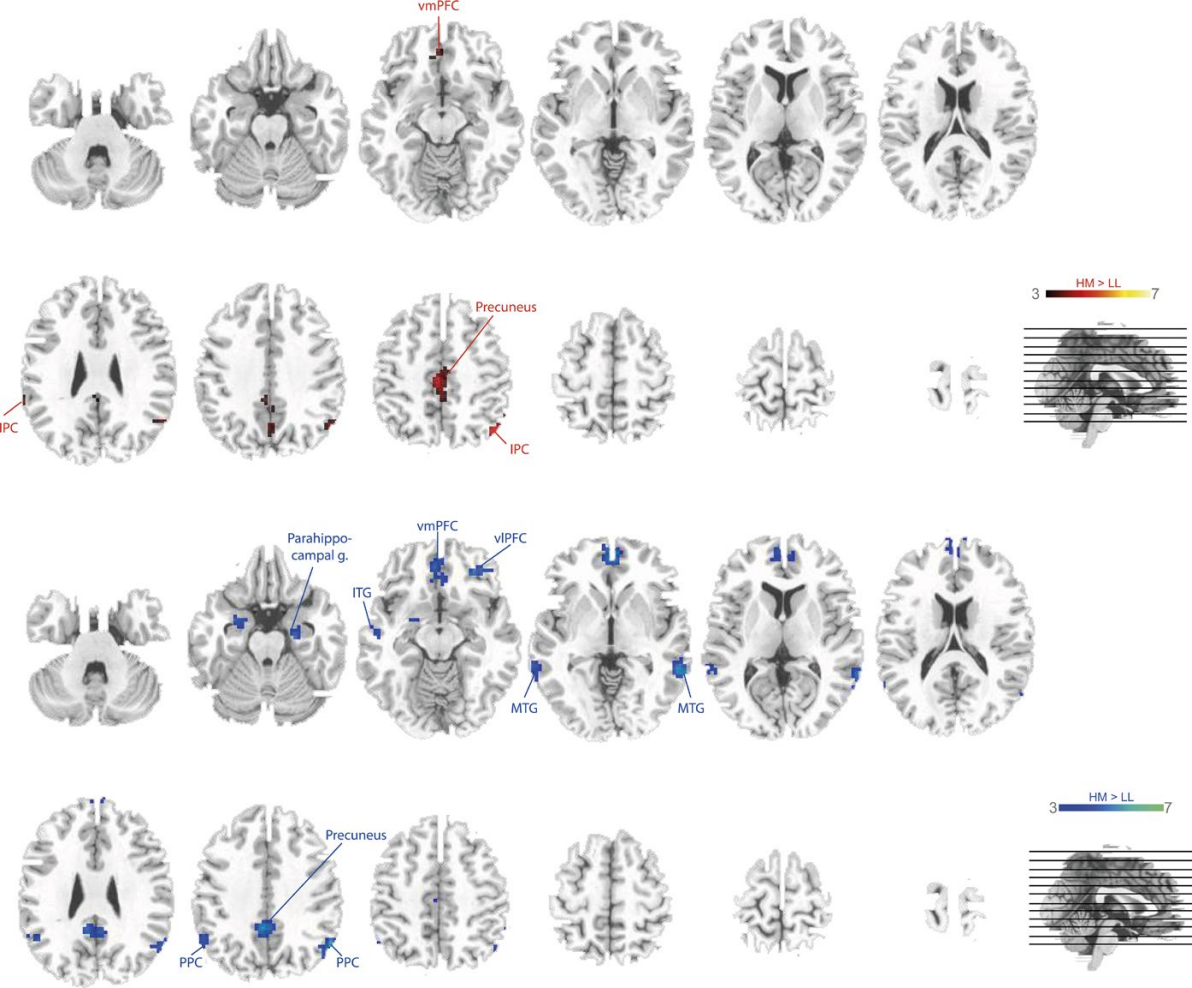
Storage contrast. Colored voxel mark a significant contrast HM > LL for young (red) and old participants (blue), *p *= .001 (uncorr.)

Brain regions involved in information storage were identified by contrasting activation of the high memory condition (HM) against the low memory condition (LL). Activations at a significance level of *p *< .001 were found in the precuneus, in the right posterior parietal cortex (rPPC) and in the left ventromedial PFC (vmPFC) (Fig. 4). A more extensive net of coactivated brain regions involved in storage of information was found in the elder participants. The aged cohort recruited the right PPC, the bilateral ventromedial and the left ventrolateral PFC, the precuneus, the bilateral middle and inferior temporal gyri (MTG and ITG), the right superior temporal gyrus (STG), the left cingulum and the bilateral parahippocampal gyrus (Fig. 4). Peaks of all clusters are reported in Table 2.

**Table 2:** Peak activations for the storage contrast (HM > LL)

Anatomical structure	Hemisphere	MNI coordinates (x, y, z)			Max. T‐value	Cluster size
Young						
PPC	R	42	−70	49	4.97	14
	R	57	−61	28	3.78	
	R	54	−52	52	3.66	
	L	−66	−43	28	3.86	19
vmPFC	L	−3	41	−14	3.65	11
Precuneus	R	3	−31	40	4.68	148
	R	3	−52	34	3.46	
	–	0	−67	34	3.70	20
Old						
PPC	R	60	−40	43	4.10	15
vmPFC	R	15	62	28	5.06	297
	L	−3	50	−5	5.63	
	L	−6	62	25	4.34	
vlPFC	L	−30	38	−14	4.75	23
Precuneus	–	0	−52	31	5.41	109
MTG	R	60	−31	1	3.89	10
	R	63	−46	1	4.37	60
	L	63	−49	−2	6.10	220
	L	−54	−61	37	5.76	
	L	−57	−67	13	3.94	
ITG	R	57	−10	−17	4.08	22
	L	−60	−19	−20	4.51	21
STG	R	54	−61	37	4.07	85
	R	60	−61	16	3.54	
Cingulum	L	−3	−25	40	4.64	17
Parahippocampal g.	R	21	−7	−20	4.64	41
	L	−24	−16	−26	4.48	20

L, left; R, right; PPC, posterior parietal cortex; vmPFC, ventromedial prefrontal cortex; MTG, middle temporal gyri; ITG, inferior temporal gyri; STG, superior temporal gyrus.

#### 3.2.3 Conjunction analysis

**Figure 5:**
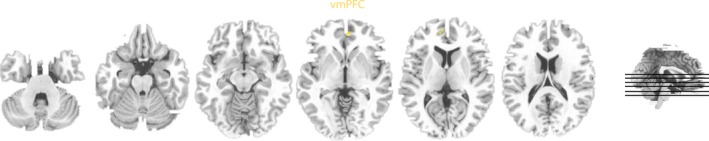
Conjunction analysis. Task‐related changes in blood oxygenation level‐dependent (BOLD) signal during encoding: Group activation map for the conjunction of the filter (HF > LL) and storage contrast (HM > LL), *p *= .001 (uncorr.) in elder

To identify brain regions that are involved during the filtering and storage of information a conjunction between the filter (HF > LL) and storage contrast (HM > LL) was calculated. No brain regions were commonly recruited in younger participants. In elder participants, the vmPFC was activated during information filtering as well as during information storage (Fig. 5, Table 3).

**Table 3:** Peak activations for the conjunction of filter (HF > LL) and storage contrast (HM > LL)

Anatomical structure	Hemisphere	MNI coordinates (x, y, z)			Max. T‐value	Cluster size
Young						
No significant clusters above a level of *p *= .001 uncorr.						
Old						
dlPFC	–	0	56	1	3.72	17
	L	−3	47	−8	3.65	10

L, left; R, right.

## 4 Discussion

The present study aimed at clarifying the neuronal basis of selective filtering and information storage within VWM with a special focus on age‐related differences. To that end, we developed a new paradigm that unlike prior ones controlled for perceptual input while filter and storage demands were modulated. Besides behavioral scores for filter and storage performance, the involved brain regions were assessed by contrasting two conditions with either high filter or high storage demands with a baseline condition, whereby the number of presented stimuli was the same in all conditions.

### 4.1 Filtering

Positive filter accuracy scores indicate that it was generally more difficult to filter out distractors based on orientation (condition HF) than on color (condition LL). However, the respective filter accuracy score did not differ between younger and older participants, suggesting that older participants’ ability to filter out irrelevant stimuli was intact. The fMRI results confirm but also go beyond the results of earlier studies (e.g., McNab & Klingberg, 2007) as a more extensive neuronal filtering network was identified in the present study. In younger participants, the filter‐related areas included the bilateral insulae, the right occipital cortex**,** the right brainstem and the right cerebellum. In addition to the bilateral insulae, elder participants recruited the left ventromedial prefrontal cortex and the right precuneus during filtering (Fig. 3). The fact that we observed a more extensive filtering network than earlier fMRI studies on this matter may be in part related to testing a larger number of participants which increased statistical power. However, one structure that previous studies (e.g., McNab & Klingberg, 2007) have also proposed as part of the filtering network did not emerge in the present study, namely the basal ganglia. But then, it is important to note that McNab and Klingberg (2007) assessed filtering during the cueing phase, that is, in the preparation phase that preceded the actual encoding process. In another study by Shulman et al. (2009), the basal ganglia among other areas were proposed to be involved in attentional shifts to locations where stimuli appeared unexpectedly. Together these findings of prior studies may point to a role of the basal ganglia in deploying attention to relevant locations BEFORE the actual encoding of the stimuli at these locations takes place. In contrast, in our study, we assessed neuronal activity AFTER the initial preparation phase and during the selective encoding of relevant and the simultaneous filtering out of irrelevant information. This situation resembles more the stimulus‐driven attentional orienting process (Corbetta & Shulman, 2002), a process known to involve the insulae which were found to be active during filtering in the present study too. The emergence of the occipital cortex during filtering in the younger group was not surprising. Attention‐driven enhancement of visual areas in occipital cortex is an often documented observation and is assumed to stem from frontoparietal networks asserting top‐down control over visual areas (e.g., Ruff, 2013). The right cerebellum was also among the brain regions recruited during filtering in the younger group. The involvement of the cerebellum in WM processes is, nowadays, generally agreed upon but its exact function, especially during information selection, is still not clear. An attempt to disentangle the role of the cerebellum during memory and filtering processes was made by Baier, Müller, and Dieterich (2014). In this study, patients with cerebellar lesions due to strokes had to perform a VWM task. The patients were only impaired in performance when targets were presented among distractors. The authors proposed the cerebellum as part of a neuronal gatekeeper network. This assumption might explain the emergence of the cerebellum during filtering in the present study.

In elder group, in addition to the insulae, the left ventromedial PFC were active when filtering demands were high which is in line with other studies reporting an increase in frontal activity during cognitively challenging tasks in healthy aging (e.g., Payer et al., 2006). Hence, the additional recruitment of frontal brain regions during filtering can be explained as functional compensation of a structural decline, that is, frontal atrophy (Raz et al., 2005). And indeed, in the present study, behavioral performance did not differ between age groups. Similarly, the activation of the precuneus which is known to be involved in information inhibition (Berron, Frühholz, & Herrmann, 2015) during challenging filtering in the elder group only might reflect a compensatory process.

### 4.2 Storage

We found the bilateral PPC, the left vmPFC and the precuneus in both age groups to be more active during trials with high memory storage but low filtering demands (Fig. 5). Similar to the filter contrast, the storage contrast revealed a much more extensive network in the elder participants. In addition to the brain regions also found in the younger group, they recruited the bilateral ventromedial and left ventrolateral PFC, the bilateral middle, superior and inferior temporal lobes, the left cingulum and the bilateral parahippocampal cortex. The finding that more frontal areas were recruited in elder than in younger participants might relate to the fact that our younger participants could largely rely on more posterior brain areas during information storage (Müller & Knight, 2006). Memory storage in the elder on the other hand seems to be sustained by a more distributed neuronal network including several ventral frontal brain areas.

The emergence of the posterior parietal cortex (PPC) during demanding memory trials in both age groups was expected and is well in line with the literature on the location of an assumed storage node in the brain (Todd & Marois, 2004, 2005; Vogel & Machizawa, 2004; Xu & Chun, 2005). The essential role of the PPC in memory was further confirmed in studies reporting memory deficits in patients with parietal lesions (e.g., Baldo & Dronkers, 2006; Finke, Bublak, & Zihl, 2006). Likewise, the precuneus has been related to storage of verbal (LaBar, Gitelman, Parrish, & Mesulam, 1999) and visuospatial information (Raabe, Fischer, Bernhardt, & Greenlee, 2013). The involvement of the temporal cortex in WM storage which was observed only in the elder group in the present study has also been reported before. For example, sustained responses to stimuli after their withdrawal were not only found in prefrontal and parietal but also in temporal cortex (Miller & Desimone, 1994). As part of the ventral pathway, the temporal cortex is known to be active during the encoding of objects (Ranganath, DeGutis, & D'Esposito, 2004). The functional role of the cingulate gyrus especially during memory, however, is not well studied. Its posterior part, which was active during memory storage in the present study, was previously reported to be involved in autobiographical episodic memory (Maddock, Garrett, & Buonocore, 2001) and during recognition of words, objects, and places (Heun et al., 2005; Sugiura, Shah, Zilles, & Fink, 2005). Furthermore, the size of the posterior cingulate gyrus was found to correlate with several parameters of a memory test including verbal and nonverbal memory capacity and errors in the visual recall of geometric objects (Kozlovskiy, Vartanov, Nikonova, Pyasik, & Velichkovsky, 2012). In addition, in the elder group, the parahippocampal cortex was modulated by memory load. This is in line with reports of the parahippocampal cortex playing a role in the encoding period of WM tasks (Olsen et al., 2009; Schon, Hasselmo, LoPresti, Tricarico, & Stern, 2004).

### 4.3 Conjunction analysis

In this study, we were also interested in brain regions that are crucial for both processes under investigation here. For that purpose, conjunction analyses were carried out for each age group across both contrasts (Fig. 5). We did not find a single brain region that was commonly activated during storage and filter processes in the younger group. In the elder group, however, the ventromedial prefrontal cortex emerged in both contrasts. Hence, this region qualifies as an interface between both processes that may adopt a control function (e.g., Corbetta & Shulman, 2002; Lepsien & Nobre, 2006; Pessoa, Kastner, & Ungerleider, 2003). The fact that this region did not emerge in younger participants might reflect that both high load tasks were more demanding for elder participants who then activated frontal brain regions in compensation. This is in line with other studies showing an activation increase in frontal brain regions when task demands are high (Payer et al., 2006; Reuter‐Lorenz & Cappell, 2008).

### 4.4 Limitations

One limitation of the present study is that fMRI results are based on an uncorrected threshold. However, while more conservative thresholds (e.g., FDR or FWE correction) reduce the number of type I errors, they at the same time increase the number of type II errors and, therefore, raise the concern that true effects are missed. This concern has led others to propose to use a combination of intensity and cluster size in order to achieve a good compromise between type I and type II errors (Lieberman and Cunningham, 2009). The authors suggested a threshold of *p *< .005 combined with a cluster size of 10 voxels to get a good balance between both types of errors. In our study, we decided to use a slightly more conservative threshold of 0.001 and a cluster size of 10 which–we believe–is a sensible approach in order to account for both error types.

## 5 Conclusion

Together, the present results allow for a more thorough insight into age‐dependent neural filter and storage networks of VWM. By testing a large sample of participants and by avoiding confounds from perceptual load, we identified new network nodes like the insulae, the occipital cortex, the brainstem, the right cerebellum, the precuneus and the ventromedial prefrontal cortex for filtering and the posterior parietal cortex, the ventromedial and ventrolateral prefrontal cortex, precuneus, temporal cortex, cingulum and parahippocampal cortex for storage. Regarding age effects, we observed generally larger network activity in our elder participants. In the elder group, increasing either selection or memory load led to the recruitment of the same prefrontal brain region. We suggest that this region exerts compensatory cognitive control mechanisms irrespective of which processes are challenged. The results further show that similar behavioral performance in different age groups can be achieved by different underlying brain processes. The usual method for evaluating interventions to improve cognitive deficits in elderly is to first test these interventions in young participants. This approach might be misleading because of the different underlying neuronal mechanisms in young and old. Understanding better the neural processes leading to cognitive deficits in healthy aging would, therefore, help in developing effective prevention programs against age‐related cognitive decline.

## Funding Information

Deutsche Forschungsgemeinschaft, (Grant/Award Number: ‘Mu1364/4‐1’,’Mu1364/4‐2’).

## Conflicts of Interest

The authors declare that there are no conflicts of interest.
